# Omics Approaches in Drug Development against Leishmaniasis: Current Scenario and Future Prospects

**DOI:** 10.3390/pathogens12010039

**Published:** 2022-12-26

**Authors:** Ali A. Rabaan, Muhammed A. Bakhrebah, Ranjan K. Mohapatra, Ramadan Abdelmoez Farahat, Manish Dhawan, Sara Alwarthan, Mohammed Aljeldah, Basim R. Al Shammari, Amal H. Al-Najjar, Mona A. Alhusayyen, Ghadeer H. Al-Absi, Yahya Aldawood, Abdulmonem A. Alsaleh, Saleh A. Alshamrani, Souad A. Almuthree, Abdulsalam Alawfi, Amer Alshengeti, Ameen S. S. Alwashmi, Khalid Hajissa, Majed S. Nassar

**Affiliations:** 1 Molecular Diagnostic Laboratory, Johns Hopkins Aramco Healthcare, Dhahran 31311, Saudi Arabia; 2 College of Medicine, Alfaisal University, Riyadh 11533, Saudi Arabia; 3 Department of Public Health and Nutrition, The University of Haripur, Haripur 22610, Pakistan; 4 Life Science and Environment Research Institute, King Abdulaziz City for Science and Technology (KACST), Riyadh 11442, Saudi Arabia; 5 Department of Chemistry, Government College of Engineering, Keonjhar 758002, India; 6 Faculty of Medicine, Kafrelsheikh University, Kafrelsheikh 33511, Egypt; 7 Department of Microbiology, Punjab Agricultural University, Ludhiana 141004, India; 8 Trafford College, Altrincham, Manchester WA14 5PQ, UK; 9 Department of Internal Medicine, College of Medicine, Imam Abdulrahman Bin Faisal University, Dammam 34212, Saudi Arabia; 10 Department of Clinical Laboratory Sciences, College of Applied Medical Sciences, University of Hafr Al Batin, Hafr Al Batin 39831, Saudi Arabia; 11 Drug & Poison Information Center, Pharmacy Department, Security Forces Hospital Program, Riyadh 11481, Saudi Arabia; 12 Pharmacy Services Department, Prince Sultan Cardiac Center, Riyadh 36441, Saudi Arabia; 13 Department of Pharmacy Practice, College of Pharmacy, Alfaisal University, Riyadh 11533, Saudi Arabia; 14 Clinical Laboratory Science Department, Mohammed Al-Mana College for Medical Sciences, Dammam 34222, Saudi Arabia; 15 Department of Clinical Laboratory Sciences, College of Applied Medical Sciences, Najran University, Najran 61441, Saudi Arabia; 16 Department of Infectious Disease, King Abdullah Medical City, Makkah 43442, Saudi Arabia; 17 Department of Pediatrics, College of Medicine, Taibah University, Al-Madinah 41491, Saudi Arabia; 18 Department of Infection Prevention and Control, Prince Mohammad Bin Abdulaziz Hospital, National Guard Health Affairs, Al-Madinah 41491, Saudi Arabia; 19 Department of Medical Laboratories, College of Applied Medical Sciences, Qassim University, Buraydah 51452, Saudi Arabia; 20 Department of Zoology, Faculty of Science and Technology, Omdurman Islamic University, Omdurman 14415, Sudan

**Keywords:** leishmaniasis, proteomics, structural proteomics, target proteins, drug discovery

## Abstract

Leishmaniasis is a zoonotic disease transmitted in humans by the bite of *Leishmania*-infected phlebotomine sandflies. Each year approximately 58,500 cases of leishmaniasis are diagnosed across the globe, with a mortality rate of nearly seven percent. There are over 20 parasitic strains of *Leishmania* which are known to cause distinct types of leishmaniasis and pose an endemic threat to humans worldwide. Therefore, it is crucial to develop potential medications and vaccines to combat leishmaniasis. However, the task of developing therapeutic solutions is challenging due to *Leishmania*’s digenetic lifecycle. The challenge is further intensified by cases of resistance against the available drugs. Owing to these challenges, the conventional drug development regimen is further limited by target discovery and ligand suitability for the targets. On the other hand, as an added advantage, the emergence of omics-based tools, such as high-end proteomics, transcriptomics and genomics, has hastened the pace of target discovery and target-based drug development. It is now becoming apparent that multi-omics convergence and an inter-connected systems approach is less time-consuming and more cost-effective for any drug-development process. This comprehensive review is an attempt to summarize the current knowledge on the muti-omics approach in drug development against leishmaniasis. In particular, it elaborates the potential target identification from secreted proteins in various stages of *Leishmania* infection and also illustrates the convergence of transcriptomic and genomic data towards the collective goal of drug discovery. This review also provides an understanding of the potential parasite’s drug targets and drug resistance characteristics of the parasite, which can be used in designing effective and specific therapeutics.

## 1. Introduction

Leishmaniasis is an infectious disease triggered by protozoan parasites spread transversely to over twenty *Leishmania* species [1,2]. Parasites of *Leishmania* are transferred *to* humans via the bite of a diseased female phlebotomine sandfly (2–3 mm long-insect vector). *Leishmania* is classified for causing three major types of infections—visceral leishmaniasis (VL), cutaneous leishmaniasis (CL) and mucocutaneous leishmaniasis (MCL), with VL being the most severe form of infection and CL the least communicative form—which are instigated through dermotropic *Leishmania* species, and allied with scarring skin lesions on the face, arms and legs [3]. Leishmaniasis is most prevalent in Middle East Asia, Southern America and South Europe [4]. Globally, it has been estimated that 70,000–1.2 million new cases of CL and fewer than 10,000 cases of VL [5] are detected each year. Unfortunately, leishmaniasis is still considered a neglected disease [6,7]. 

**Figure 1:**
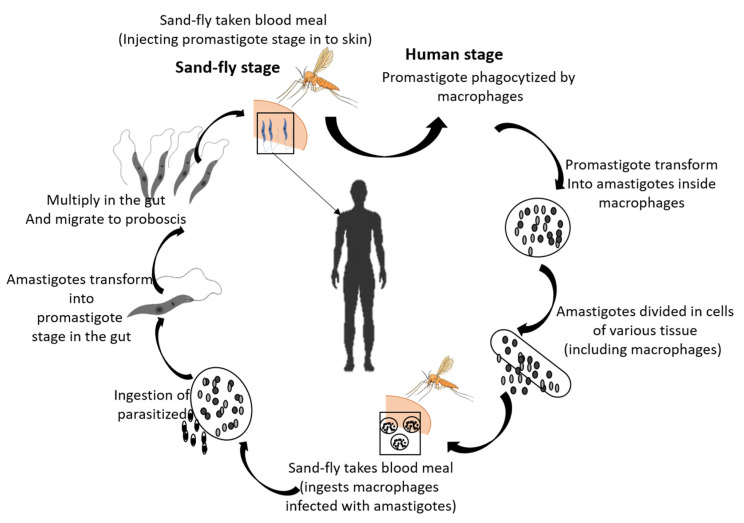
*Leishmania* is transferred from the bite of the female sandfly. (1) Through blood meals, sandflies infuse the transferable stage, promastigotes. (2) Promastigotes are phagocytized by macrophages when they reach to puncture the wound and are converted into amastigotes. (3) Amastigotes are divided into infected cells and distress numerous tissues. (4) Clinical Leishmania symptoms begin when the sandfly ingests the amastigote. (5) The transmission cycle is completed when an amastigote converts into a promastigote in the sandfly gut.

The *Leishmania* lifecycle, shown in Figure 1, is activated when an infected sandfly bite inoculates parasites in their promastigote form into the skin of a mammalian host [8,9]. Later the promastigote is phagocytized by macrophages and transforms into an amastigote, which multiplies in cells and is transmitted to other cells [3,10]. Leishmaniasis is diagnosed through light microscopic examination of skin lesions and molecular methods such as PCR that are used to differentiate the species of *Leishmania* [11,12,13]. Treatment of leishmaniasis depends upon the type of species. In the case of cutaneous leishmaniasis (CL), pentamidine and amphotericin B are the group 2 drugs that are used to reduce the symptoms of CL. They need a long course of intravenous administration [14,15]. Another optimal treatment is pentavalent antimonial therapy (Sbv) [16,17]. It has a double mode of action: one mode is the disruption of the parasites’ redox balance, while the second is the imposition of additional oxidative and nitrosative stress via interaction with the host cell [10]. Oral treatments that are approved for this disease include miltefosine and the azole antifungal compounds [18,19,20]. However, the aforementioned treatments are confronted with several limitations, such as non-universal nature as well as side effects [21,22]. It has been shown in some studies that miltefosine cannot be used to treat pregnant women and that long-term use of azole as a potential treatment is accompanied with hepatotoxicity and hormone-related side effects. Some commonly reported side effects under hormonal dysregulation include decreased libido, gynecomastia, alopecia, impotence, hyponatremia, azoospermia, hypokalemia, oligospermia and (rarely) adrenal insufficiency [23,24,25]. Therefore, it is necessary to develop novel therapeutics for this disease. Proteomics offers an excellent choice to cope with these challenges and help us to understand the host pathogen interaction in leishmaniasis; this has sped up the discovery of a new and more effective therapeutic against leishmaniasis [26,27,28].

**Figure 2:**
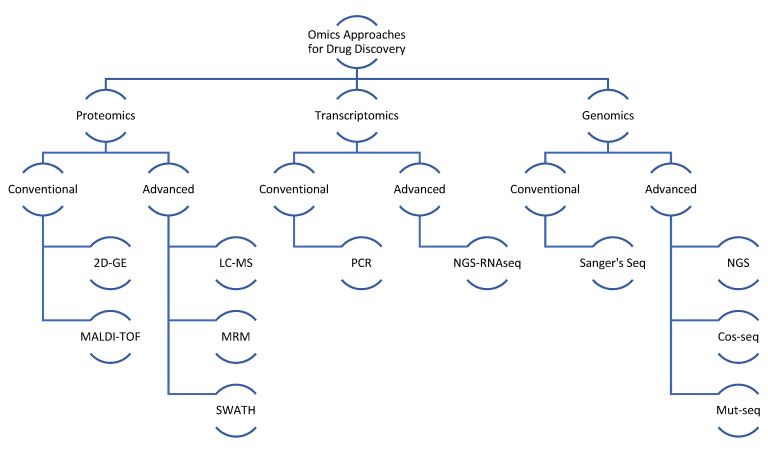
Omics analysis methods used in the study of *Leishmania* parasite.

Proteomics plays an essential role in biomedical research to understand the protein–host interaction and develop new and effective drugs against various diseases [29,30]. Proteins are the functional units of the cell, and the dynamic nature of protein profiles highlights the significance of studying protein expression as a factor in elucidating intricate cellular behavior [31]. Deciphering protein functions is still based on the structural mapping of the proteins. Therefore, proteomics technology advancements have resulted in the occurrence of several new aspects of proteomics, including structural, functional and expression data of proteomics. Here, comprehensive proteomics studies are applied to understand the systematic structure of proteins and their functions. Structural proteomics provides a foundation for comprehending protein production through high-throughput cloning and expression from multiple vectors [26,27]. Several biomedical fields, including medicine and dentistry, have been revolutionized by the use of proteomics tools. Proteomics has made significant contributions to the field of dentistry by aiding in the identification of various biomarkers present in oral fluids for the early diagnosis of several diseases. Additionally, proteomics has contributed to the identification of several medically significant biomarkers for various diseases [26]. Numerous methods shown in Figure 2 are used in protein analysis that involves mass spectrometry (MS), sodium dodecyl sulfate polyacrylamide gel electrophoresis (SDS-page), 2D-gel electrophoresis (2DGE) and high-performance liquid chromatography (HPLC). In these approaches, the most commonly used techniques in proteomics are 2DGE combined with MS. 2DGE isolates a complex mixture of proteins by using two different properties of the proteins, the pI value in the first dimension and the relative molecular weight in the second dimension. The primary strength of 2DGE is that it can detect >10,000 protein spots in a single gel run, even though it is characterized by post-translational modification by changes in protein charge, which can be easily observed by an altered pI value. However, there are some limitations to 2DGE. The major limitations of the 2DGE approach are its inability to resolve very hydrophobic proteins with extreme pI values and its poor dynamic ranges that mean it cannot detect all levels of proteins and molecular weight simultaneously. Another is its inability to resolve proteins which are too basic, too acidic, too large or too small; however, this limitation has been continuously decreased with the advancements in proteomics technology.

The limitations of the classical approaches of proteins have recently begun to be remedied by advanced methods such as mass spectrometry. The mass spectrometric methods are primarily based on the fundamental of fragmentation of peptides and imparting uniform charge, which are later separated using mass analyzers; the spectra thus obtained are used for the further identification of peptides. Although mass spectrometry-based methods initially gained their popularity for global proteomics and hence expression analysis, the ability to tag proteins using isobaric tags or the tagging of amino acids made the quantitative analysis easier and much more comprehensive. These methods were later sub-classified as quantitative proteomics. Over the past decade, proteomics has added more approaches to its domain, including targeted proteomics approaches and reaction monitoring methods. Selected ion monitoring (SIM), selective reaction monitoring (SRM) and Multiple reaction monitoring (MRM) have emerged as powerful tools in proteomics. These methods have an added advantage over conventional proteomics in terms of sensitivity. Moreover, these methods are considered superior for peptide identification in complex biological samples such as human plasma and serum, hence providing ample opportunities for drug-target identification with higher probability and success rates [32]. Additionally, reaction kinetics, which forms an essential component of the drug validation and assessment of draggability, can also be performed using mass spectrometry. Mass spectrometry can be used as the readout for characterizing the kinetics of rapid reactions, but it has been limited to millisecond time resolution [33]. The innovative SWATH-MS (Sequential Windowed Acquisition of All Theoretical Mass Spectra) approach is advantageous to several other contemporary mass spectrometry-based methods due to its strength, data-independent acquisition (DIA) and high sensitivity. Compared to gel-based or label-based methods, protein SWATHE provides quantitative consistency and accuracy [34]. Nevertheless, these methodological advancements have only become possible due to technical advancements in proteomics, including enhancement in mass analyzers and detectors from conventional time of flight analyzers to quadrupole and orbitrap modes. 

Besides proteomics, transcriptomics and genomics are highly valuable in augmenting the drug discovery process. Gene and RNA expression profiles also provide important information about possible targets, in particular the unique DNA features in genome or differentially expressed RNA molecules. Much of the information of transcriptomics is referred at protein level for the identification of targets, but emerging sequencing methods have also allowed use of nucleic acids as potential drug targets or ligands. Small RNAs, long-noncoding RNA, fingerprint regions and regulatory regions are all important components of nucleic acids that are useful in the drug discovery process. Earlier, when conventional approaches (Sanger’s method) for sequencing were used, the time and cost of sequencing was higher, but the advent of next generation sequencing has brought about a revolution in sequencing techniques. Among the most popular NGS platforms are illumine high seq or similar versions which provide huge data at relatively low cost, augmenting another main arm of omics for drug discovery. 

## 2. General Proteomic Approach in Different Type of Leishmaniasis

The study of the proteome of parasites in the examination of novel drugs has been the priority of the medical and scientific domain since the early 1970s and 1980s. One-dimensional electrophoresis and Edman sequencing have been developed for structural and molecular protein characterization [35,36]. In *Leishmania*, proteomic studies show the identification of biomolecules and the pathways that are involved in host–pathogen interaction in the parasites. Additionally, proteomics helps in the identification of targets for prophylactic treatments, chemotherapeutic treatments and biomarkers that are used in the diagnosis of *Leishmania* [26]. Moreover, leishmaniasis includes the alteration of protein expression upon infection. Therefore, proteomics studies are used during disease manifestations to provide information regarding the protein signature of the disease and authentication for disease intrusion. The tools used in proteomics studies also help to recognize protein markers to screen their efficacy for diagnosis and clinical prosecution [26]. Moreover, in several studies of proteomic analysis, various drug-targeted proteins against leishmaniasis are described in Table 1. Therefore, in this section of the review, we attempt to discuss assorted studies on selected and targeted proteins against *Leishmania* species and the type of leishmaniasis disease associated with them. 

Visceral leishmaniasis (VL) is predominantly caused by *Leishmania donovani* and *Leishmania infantum*. In the Mediterranean region it is caused by *L. infantum* and is known as Mediterranean visceral leishmaniasis (MVL). In the case of MVL, the dog is the natural host, while the human is an accidental host as a sandfly has bitten the infected dog which has later transmitted it to humans. VL in humans vary and sometimes it appears as life-threatening disease. VL is diagnosed by various parasitological and molecular methods. Moreover, Th1 cell is accountable for immunity against Visceral leishmaniasis. Th1 is a subset of T cells that produces IFN-𝛾, which activates macrophages to kill parasites. However, most treated patients demonstrated resistance to an infection-related development of the Th1 cell antigenic response. As a result, studies on Th1 cells against the *Leishmania* parasite should be prioritized in the development of a vaccine against visceral leishmaniasis. Presently, new techniques such as proteomics offer a promising strategy for identifying new vaccine targets for leishmaniasis. Techniques involved in proteomic analysis are 2D gel electrophoresis, protein elution and tryptic digestion, HPLC/MS, and protein identification by using a protein database [37].

In their proteomics-based drug discovery strategy, Gupta and his co-workers targeted visceral leishmaniasis caused by *Leishmania donovani*. In this study, a soluble protein from the promastigote form of *L*. *donovani* (clinical isolate) with a molecular weight range of 68 to 97.4 kDa (F2 fraction) induced a Th1 response in the peripheral blood mononuclear cells of cured *Leishmania* patients, indicating that it possesses significant prophylactic potential. Several proteomics techniques, such as 2DE, MALDI-TOF and MALDI-TOF/TOF-MS, were employed to comprehend the nature of the f2 proteins. In this analysis, 63 resolved proteins were detected and 33 proteins, including six unknown proteins, were identified. Known immunostimulatory or immunogenic proteins, such as elongation factor 2, p45, heat shock protein (HSP) 70, HSP83, fructose 1–6 biphosphatealdolase, enolase, triosephosphate isomerase, protein disulfideisomerase, calreticulin and kinesin-like protein, were evaluated as potential drug targets. Consequently, this study demonstrates the value of proteomics in the characterization of a complex protein fraction (F2) map of a soluble *L*. *donovani* promastigote antigen identified as Th1 stimulatory for its potential as a vaccine target against VL [38]. 

Another leishmaniasis disease is cutaneous leishmaniasis (CL). It is the most commonly appearing leishmaniasis, and it leaves permanent scars and lesions on the skin. The current drugs used to treat CL have various flaws concerning safety, resistance and availability. Th1 and CD4^+^ show immunity against the CL treatments [39,40]. Moreover, several proteomics studies were done to analyse and identify potential proteins and biomarkers for the treatment of CL. In addition, previous researchers performed a label free quantitative proteomic approach to identify the potential biomarkers for the early detection of CL, which is caused by *Leishmania panamensis*. In this study they compared the proteomic profiles of patients with CL before and after treatment. While in the proteomic analysis, 12 protein expressed differently from while in the comparative LC-electrospray ionization MS/MS (LC-ESI-MS/MS) triplicate analysis, in which seven proteins (KRT73, LAMP2, S100A9, S100A8, KRT10, SERPINB1 and ATP7A) were found as up-regulated and five (FABP5, S100A2, PRDX2, HBA1 and HBD) were found as down-regulated. Moreover, all differentially expressed proteins were classified according to their molecular function, biological function and class; from them calcium binding protein A2, A8, A9 and haemoglobin subunit alpha-2 and delta exhibited a high correlation with epidermis development and immune response. It was thus concluded that the differentially expressed proteins were associated with inflammation reduction and enhancement of tissue repair. Therefore, these proteins can be used as potential biomarkers for the early detection of CL [41].

Wright and his co-workers focused on the exploration of N-myristoyltransferase (NMT) as a drug target in the *Leishmania donovani.* However, only a small number of NMT substrates were observed in *Leishmania.* In this study lipidated proteins in the promastigote and amastigote life stages were identified by metabolic tagging (YnMyr) with an alkyne-functionalized myristic acid mimic in live parasites, followed by click chemistry. Metabolic tagging with YnMyr in the amastigote stage found a distinct labelling pattern compared with the promastigote stage and no diffuse band. In addition, quantitative chemical proteomics using mass spectrometry was performed to characterize the interaction of targets by NMT inhibitors, to identify the complement of *N*-myristoylated proteins in *L*. *donavani* parasites, and to explore the NMT as a potential drug target for *L. donavani*. These findings predict the pleiotropic effects of NMT inhibition in parasites and offer methods for testing targeted interactions in the cell [42]. 

Corpas Lopez and his co-workers’ study centred on the pharmacological affirmation of *N*-myristoyltransferase as a drug target in *Leishmania donovani*. In this study 1600 pyrazolyl sulfonamide compound was screened against *L. major* NMT by high-throughput screening assay. Here, several potent inhibitors with marginal selectivity over the human enzyme were identified, while the potent inhibitor DDD85646 of NMT in *Tropanosoma brucei* was efficient in treating animal models of stage 1 but not stage 2. This led to the discovery of DDD100097, a potent TbNMT inhibitor with moderate efficacy in a mouse model of Stage 2 disease. In addition, the most effective inhibitors of *Leishmania* NMT (Lm NMT) were the methylpiperidine analogues 2 and 3, which exhibited 10-fold selectivity over the human enzyme. Later, *L. donovani* axenic amastigotes were screened with the most enticing LmNMT inhibitors. These two independent techniques, screening against their cosmid-based overexpression library and thermal proteome profiling (TPP), demonstrated that DDD100097 (compound 2) functions as designed within parasites. Furthermore, in their mouse model of visceral leishmaniasis, administration of compound 2 reduced parasite burden by 52%. Consequently, NMT is now a validated pharmacological target for *Leishmania* [43].

In another study, Dashatan and his colleagues used topological analysis of protein–protein interactions to identify key proteins against *Leishmania major* through the bioinformatics approach. During the examination, they extracted 252 related proteins from UniProt related to *Leishmania major*. Then the protein network of these proteins was explored and visualized using the STRING database and Cytoscope software version 3.0.2. Subsequently, gene ontology was performed using the MCODE algorithm, which was carried out by Protein Analysis Through Evolutionary Relationships (PANTHER) databases in order to obtain relevant results. This study identified the hub proteins UB-EP52, EF-2, chaperonin, HSP-70.4, beta chain, tubulin alpha LACK and ENOL as potential proteins in understanding the pathophysiology of *Leishmania major*, resulting in disease control as well as a strong possibility of designing a new drug through these hub proteins [44]. 

Using microarray facts and applying in silico methods, *Leishmania species* miltefosine (MIL) resistance was identified by Lari and his co-workers. In this study, they extracted GSE30685 and GSE45496 from the gene expression omnibus (GEO) database and analysed them with the GEO2R application to detect genes involved in MIL-resistant *Leishmania species*. Here, 177 differently expressed genes (DEGs) were chosen from the GSEs, 50% of which were uncharacterized proteins. Further, the STRING database and PPI networks were used to analyze the interactions between DEGs. The PPI network was found to contain five hub proteins (*LDBPK_220630*, *LDBPK_282140*, *LDBPK_210210*, *LDBPK_291190*, and *LDBPK_15017*) according to the study. Protein LDBPK_150170 encoded PP2C was found to be more active against MIL-resistant *Leishmania major* parasites. Moreover, *Leishmania major* miltefosine transporter (LmMT) markers and small hydrophilic endoplasmic reticulum-associated protein (SHERP) genes were also detected in Iranian MIL-resistant *L. major* parasites. Therefore, these data recommended PP2C, SHERP and LmMT as the potential drug objects for MIL-resistant *Leishmania major* therapy [45].

Florez and his co-workers predicted the protein interface network of *Leishmania major* employing PSIMAP, PEIMAP and ipfam. The results of these approaches were combined and calculated into a network with 1366 nodes and 33,861 interactions and a high degree of confidence. Later, enrichment analysis on the detected clusters was performed. This predicted the biological processes of 263 interacting proteins. There were 91 essential kinases predicted to lack homology with the human kinome. They were treated as potential leishmaniasis drug targets. In addition, 142 drug targets (UniProt id of some targeted proteins out of 142: Q4QFA8, Q4QIR9, Q4QH47, Q4FWM4, Q4Q9P0, Q4QCC1, Q4FYE1) were identified by analysing the topology of networks with metrics such as connectivity and betweenness centrality, after homology filtering with the human proteome [46,47]. 

In another study, Krobthong and his co-workers identified drug target proteins for *Leishmania orientalis*, *L. martiniqueensis* and *L. donovani* capitalizing proteomics data. During the proteome analysis, 6099 proteins were identified, of which 1065 were used for analysis. In this study, 16 proteins were identified as therapeutic targets for all species, and an in silico analysis of protein antigenicity showed that eight proteins (E8NH10, A4IAU0, A0A640KJ19, A0A451EJM4, ndpk, A0A3S7WPD8, A4HEA3, Q4Q8G4) genes hydrophferently conveyed Pjorlar amastiogote have the potential for the development of antigenic molecules for the treatment of diseases caused by any Leishmania species [48].

## 3. Proteomic Analysis of Different Leishmania Species

*Leishmania* is transmitted through the bite of a female sandfly that belongs to the genus Phlebotomus. In sandflies, it promotes the development of *Leishmania* and transmits it through host inflammasome–mediated interleukin (IL)-1beta that amplifies neutrophil recruitment. Neutrophils are central to acute inflammation caused by bite specific mediators, but dermal resident macrophages are gaining attention as participants that harbor *Leishmania* parasite infection [49,50]. *Leishmania* parasites are present in two categories. These are promastigote, a flagellated type possessed by sandflies, and amastigote, a non-flagellated form found in the phagosomes of macrophages in mammalian hosts. When a diseased sandfly bites the host, it transmits in a promastigote form that enters phagocytic cells. Within the phagocytic cell, it transforms into amastigotes, and the replicative amastigotes go into the sandfly while feeding the infected host. The amastigotes inside the sandfly undergo various transformations, with non-infected stages. In the primary stage, it develops a procyclic promastigote that converts into an elongated motile nectomonad promastigote, and then it transforms into a small replicative form, the leptomonad promastigote. This allows flies to start a second growth cycle in the anterior midgut and prepare to re-infect fresh phagocytes [8,51,52]. Moreover, amastigotes are the intracellular form of the parasite; information gained from these proteins has contributed significantly to the enhancement of resources for the identification of therapeutic targets and represents a promising field for the discovery of new pathogenic markers [53].

**Figure 3:**
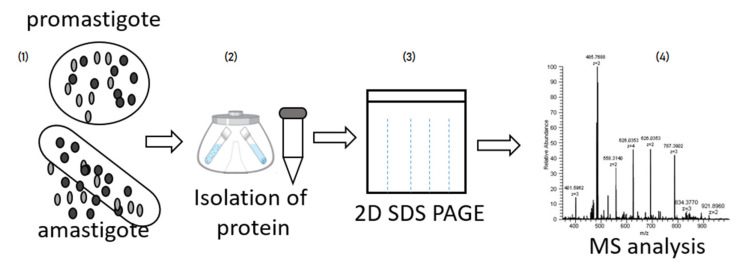
Simplified proteomics workflow of Leishmania proteome: (1) In vitro culture of *Leishmania spp*. in a medium with promastigote and amastigote forms followed by aliquoting; (2) Centrifugation of the culture and sediment collection; (3) Separation of individual proteins/subunits using 2D-Gel electrophoresis; and (4) deification of individual spots (proteins/subunits) using mass spectrometry.

Different proteins can be identified in the development stages of *Leishmania* species as shown in Figure 3.

Lynn and his co-workers performed a differential quantitative proteomic approach on *Leishmania infantum* and *Leishmania mexicana.* In this study quantitative mass spectrometry with iTRAQ labeling was used for proteomic analysis in the promastigote and amastigote life forms of the species, in which 189 and 107 proteins in *L. infantum* and *L. mexicana* were spotted, respectively. From them 20–405 proteins showed differential expression levels among the promastigote and amastigote life stages. These differentially expressed proteins mapped to various pathways, including cell motility, metabolism and infectivity. In addition, virulence factors (eEF-1, amastin, and leishmanolysin (GP63)) also play a significant role in the parasite invasion and host parasite interaction or parasite survival, and therefore may possess therapeutic potential as drug targets [54]. The protein list is given in Supplementary Tables S1 and S2. McNicoll and his co-workers used an inclusive approach; this consists of prefractionation of proteins and global proteomics and targeted microarray investigation to study the different *Leishmania* stages and their proteins by excluding a few abundant structural proteins to minimize the complexity. Here, they found various novel contrarily expressed protein isoforms in *L. infantum.* Furthermore, by performing 2D gel electrophoresis, more than 2200 isoforms of protein were found in each developmental stage. In this study, 6.1% of novel proteins appeared in the promastigote stage, while 12.4% were observed in the amastigote stage [55]. The protein list is given in Supplementary Table S3.

*Leishmania braziliensis* is responsible for most of the human tegumentary leishmaniasis (HTL) and has caused a wide range of clinical expressions, which include cutaneous leishmaniasis (CL) and mucosal leishmaniasis (ML) [56,57]. In addition, the study conducted by Curevo and his co-workers describes the proteomic analysis of *Leishmania braziliensis* by 2D gel electrophoresis and mass spectroscopy for the identification of expressed proteins in *Leishmania braziliensis*. As a result, they obtained 20 hypothetical proteins, which were identified and divided into 15 groups according to the biological process. 40% of the identified proteins were discovered for the first time in the *Leishmania* proteomic map. Moreover, various drug targets and virulence factors were identified in the protein map, of which some proteins are associated with the metastatic phenotype. This study also represents the first proteomic reference map for *L*. *braziliensis* (p*I* 4-7, M_r_ 10-130 kDa) protein by using promastigote forms of the IOC-L2643 strain, which is isolated from the mucosal disease patient. Additional research into the potential role of EF-1 as a factor involved in parasite dissemination could be of particular interest. A comparative proteomic study of the parasite with other *Leishmania* strains isolated from patients presenting distinct manifestations could help with understanding the relationship that exists between EF-1 and the biological behavior of this parasite [57]. Another study by Garcia and his co-workers focused on the new target employed in the diagnosis of HTL. Here, initially, 2D gel electrophoresis followed by MS was used to identify the proteins expressed in the amastigote and promastigote stages in conjunction with heat map analysis and an immunoproteomics approach. Eight proteins were identified in the amastigote stage of the *L*. *braziliensis* and similarly found in CL and ML patients’ samples. Later, a chimeric protein was designed based on the combination of thirteen linear B-cell epitopes identified by immunoinformatics from *L. braziliensis* proteins. As a result, it was observed that the strategy used in this work was helpful in developing an antigen for use in an immunological assay for the detection of HTL cases in comparison with results obtained from the ELISA and immunofluorescence assay [56]. 

Leifso and colleagues reported 8160 genes that were identified through DNA oligonucleotide genome microarrays by analyzing the mRNA expression profiles of *L. major* promastigotes and lesions identified in the amastigote stage. This implied that 94% of genes were articulated during both developmental stages, whereas, in statistical exploration, a low amount of mRNA manifestation was observed at 1.4% total gene in amastigotes and 1.5% total gene in promastigotes of *L. major.* Furthermore, to recognize expressed proteins in the amastigote and promastigote phases of *L. infantum* isotope coded affinity tag (ICAT), a quantitative proteomic approach followed by mass spectroscopy was applied. ICAT is a chemical labelling technique that labels proteins with chemical reagents. Later, the mass-to-charge ratio of proteins can be determined using a mass spectrometer (MS). In this study, strong cation exchange chromatography was used followed by MS in which the labelling protein ratio can be accurately determined from the relative mass spectrometric signal intensities of the heavy and normal forms of labelled protein. Therefore, both quantification and identification were achieved in a single step. Following this analysis, 91 different proteins were identified, where 8/100 were expressed uniquely in the amastigote stage, 20/100 were expressed uniquely in the promastigote stage, and 72/100 were deliberated to be articulated integrally. This concluded that *Leishmania* mRNA is constitutively expressed in the promastigote and amastigote stages [58,59]. The protein list is given in Supplementary Table S4.

Ashrafmansouri and his co-workers performed quantitative proteomic analysis, and distinctly expressed proteins were identified in the axenic amastigote stage of two *Leishmania* species identified using the ion spectra MS method. This resulted in the detection of a total of 51 up/down-regulated differently expressed proteins (DEPs) in the axenic amastigote stage of *Leishmania major* and *Leishmania tropica.* In this study, 34 and 17 proteins, respectively, were discovered to be up regulated in *L. major* and *L. tropica*. During the translation process the polyadenylate-binding protein is highly regulated in *L. major* compared to *L. tropica*. Putative heat shock 70-related protein-1, mitochondrial, as a response to stress protein folding member, is upregulated less in *L. major* amastigotes than in *L. tropica* amastigotes. Protein phosphotransferase and putative 60s ribosomal protein L35 were found to be more and less regulated in *L. tropica* compared to *L. major*. The DEPs were classified into eight and four major classes of *L. major* and *L. tropica*, respectively, based on their biological processes. The majority of up-regulated proteins in *L. major* were associated with metabolic processess (41%) and translation (29%) and included 12 and 10 genes, respectively. Membrane proteins (3% of the total) represented the smallest group in this categorization. Among up-regulated proteins in *L. tropica*, the metabolic process (41%, including seven genes) and translation (35%, including six genes) showed the highest proportion. Moreover, metabolic processes and translation were identified as the most regulated categories in *L. tropica* and *L major*. Similarly, KEGG pathway analysis showed the carbon metabolism and metabolic pathways as the top pathways in the highly regulated proteins in both the *Leishmania* species. In this study, novel differently expressed proteins were found that assisted in understanding the molecular mechanism of pathogenesis and helped to identify potential drug targets [60,61,62]. The protein list is given in Supplementary Tables S5 and S6.

In another study, Fialho Junior and his co-workers determined the proteins implicated in the virulence of the amastigote and promastigote stages of *L. infantum*. Later, isobaric mass tag labelling, gel electrophoresis and mass spectroscopy were employed. In the most contagious strain of *L. infantum*, 46 proteins were downregulated and 96 were upregulated in comparison to the less virulent strain. The virulent BH400 strain expressed a larger number of upregulated proteins in both amastigote and promastigote types than the less virulent BH46 strain. The most virulent amastigote strain was rich in glycolysis-related, ribosomal and heat-shock proteins. At the promastigote stage, heat shock, stress response and ribosomal proteins are present [63,64]. The protein list is given in Supplementary Tables S7 and S8.

Applying 2D gel electrophoresis (2DGE) and bioinformatics, the protein content of the amastigote stage of Iranian *Leishmania major* and *Leishmania tropica* was compared by Ashrafmansouri and his co-workers, During 2016–2017, this study was conducted on clinically suspected cases of cutaneous leishmaniasis (CL) referred to health centres in Mashhad, Gonbad, Kerman, Shiraz and Tehran. The Ethics committee of Shahid Beheshti University of Medical Sciences approved this research. All participants provided their informed consent formally. PCR was used to amplify the amastigote form of *Leishmania* strains *L tropica* and *L. major* converted in vitro into promastigote form. Iranian *L. tropica* and *L. major* isolates’ whole proteomes in amastigote-like form were separated using the 2-DGE method, in which 354 proteins were observed in the amastigote phase for both *Leishmania* species strains, and in the comparative analysis 74 proteins were observed to be differently exhibited in *L. tropica* while 99 were observed in *L. major*. Furthermore, *L. tropica and L. major* contained 16 and 20 distinct proteins, respectively. It was concluded that the levels of protein expression in the amastigote form of *L. tropica* and *L. major* strains were distinct. It was found that the use of proteomics tools, statistical analysis of *Leishmania’s* proteome in Iranian isolates, and comparison of their protein expression levels could aid researchers in developing a more accurate diagnosis and treatment for the disease [65].

## 4. Proteomic Approach to Host Cell Constituents and Their Relationship with the Immune Response

### 4.1. Role of Macrophages

Macrophages are mononuclear cells with the ability to phagocytose. In addition, they play a crucial role in immunity and various immune responses. They exhibit defense mechanisms against various microorganisms and parasites [66]. In the case of parasites specifically, *Leishmania* plays various roles, including those of host cells and parasites. Macrophages, the primary host cells, produce cytokines and chemokines, consequently triggering incidents that relate to the intervention of the host immune response and, subsequently, to the emergence of infection or, conversely, to control of the disease. *Leishmania* disrupts vital metabolic and signalling pathways to promote parasite development. *Leishmania* also induces DNA methylation to silence microbicidal pathway-regulating genes. These novel findings illuminate *Leishmania*’s multifaceted subversion of macrophage function [67]. Therefore, in this section, we discuss the *Leishmania*-macrophage interaction studies to understand the presence of proteins and macrophage survival in *Leishmania.* Menezes and his co-workers described the medium of macrophages from the CBA strain as being resistant to *L. major* and unaffected by *L. amazonensis.* This indicated that these cells might play a crucial function in determining the results of a *Leishmania* infection. Applying proteomics approaches, they demonstrated that distinct parasite species regulate proteins associated with cell metabolism during the primary stages of *Leishmania*–macrophage interactions. Based on this study, it was speculated that differently expressed proteins show a crucial role in determining the progression of an infection [68].

Verma and his co-workers attempted to categorize membrane proteins of *Leishmania donovani* and macrophage hosts while interacting with each other through the 2DE/MALDI-TOF/MS technique. In this study, they acknowledged membrane proteins that involve stimulated protein peroxidoxin, C kinase, cytochrome C oxidase and small myristoylated protein 1 (SMP-1) from the parasite, β-actin from macrophages, and filamin A interacting protein 1 (FILIP1). Later in this study, parasite replication and persistence in macrophages were investigated, followed by a macrophage–amastigote model which was conducted in the presence or absence of the C kinase inhibitor withaferin (WA). C kinase activation was inhibited. WA decreased the replication of *L. donavani* inside the host-macrophages. This study showed the importance of protein interactions in macrophages to understand the infection formation in a host that can be targeted further for new chemotherapeutics against *Leishmania* [69].

Smirlis and his co-workers focused on a quantitative proteomics approach that modulates the pleiotropicphenotype in principle murine macrophages contaminated with the protozoan pathogen *Leishmania donovani*. In this experiment, BALB/c macrophages derived from primary bone marrow were infected with *Leishmania donovani* at the promastigote stage for 72 h, and a SILAC-based quantitative proteomic approach was used to observe proteomic changes. Furthermore, the proteome was analysed by gel electrophoresis (GEL) and strong anion exchange (SAX) fractions that revealed 6189 proteins, whereas 86 differently modulated proteins showed a response to *Leishmania donavani* infection. This proteomic analysis showed that intracellular *Leishmania donavani* directed the variation in numerous biological processes of the host cell that involve primary metabolism and catabolic processes, with an important enhancement in lysosomal organization. The study concluded that a new host–pathogen interaction mechanism was sourced from the proteome of the first macrophage infected with *Leishmania donoavani* [70].

In another study, Salotra and his co-workers focused on identifying host and parasite source membrane proteins during *L. donavani* and macrophage interaction. *L. donavani* and THP-1 macrophages were analysed using sonication and ultracentrifugation to isolate membrane proteins. Membrane proteins involved in the interaction between the host and parasite were isolated and resoluted through 2D gel electrophoresis with nominal streaking. In the pH range of 4–7, 20 major parasite spots and 10 macrophage membrane fraction spots were observed. Later, the interaction between macrophages and parasites was calculated in the presence of Withaferin (an inhibitory drug) in two distinct macrophage cell lines, THP-1 and RAW264.7. Consequently, during parasite interaction, small myristoylated protein 1 (SMP-1), peroxidoxin, activated C kinase and cytochrome C oxidase were found in the parasite, and beta actin was detected in the macrophage. These identified proteins could be investigated as possible drug targets for the cure of *Leishmania* caused by *L. donavani* [71].

### 4.2. Role of Neutrophils

In the early stage of *Leishmania* infection, neutrophils are the first cells recruited rapidly and in large numbers to the spot of a *Leishmania* infection, where they release neutrophil extracellular traps (NET), cytokines and chemokines, among others [72]. They also generate CC-chemokine ligand 3 (CCL-3) immediately after leishmaniasis infection, and this chemokine promotes the recruitment of macrophages and dendritic cells to the site of infection, which are involved in the phagocytosis of apoptotic infected neutrophils [73]. However, when *Leishmania* come into contact with other microbes and molecules produced by arthropod vectors including sandflies, they can modify the behavior of neutrophils, causing sudden lysis to extend their life. Moreover, in the long course of co-evolution, various microorganisms developed protection against the neutrophil effector mechanism and take advantage of neutrophil clearance pathways to promote the spread of *Leishmania* in the host body. Diverse microorganisms developed resistance to the neutrophil effector mechanism over the course of co-evolution and exploit neutrophil clearance pathways to promote the spread of *Leishmania* in the host body [74].

### 4.3. Role of Lymphocytes

Lymphocytes play a key role in protection against *Leishmania* infection. However, their role in pathogenesis is unknown [75]. T cell plays a key role in generating specific memory cells to characterize infections such as *Leishmania* [76]. The proteomic and biological process study conducted by Silva Santos and his co-workers analyzed the potential biological processes and elements manifest in the identified proteins of biopsies from cutaneous leishmaniasis (CL) patients infected with *Leishmania braziliensis* versus normal skin. In the study, they observed 59 differentially expressed proteins between the infected and normal skin. Moreover, assessment of biological networks using identified proteins (KIL, KPNA1, CDK11A, and CASP-9), cell adhesion (BCAM, SPECC1, and DCHS2), cell cycle (CDK11A, NEK11, HAUS5, ANKLE2, and CENP-E), immune response (TRB, KIR2DL4, IL12RB1, and GNL1) and homeostasis (SLC8A1 and ATP1A) revealed the existence of networks that may be implicated in cytotoxic T lymphocyte-mediated cell death. Later, immunohistochemical analysis was performed. Here, the expression of caspase-3, caspase-9 and granzyme B in CL patients’ tissues was validated and found to be positively correlated with lesion size. In addition, On the inflammatory site of CL patients, proteins not previously described were observed to be expressed. Therefore, the study provides a foundation for future pathogenesis research [77]. 

## 5. Proteomic Approach to Secreted Proteins in Leishmaniasis

Secreted proteins are one of the most essential protein classes. They comprise approximately one-tenth of the human genome. The systematic use of secreted proteins in assay enables signalling pathways, blood coagulation, immune defence and digestive system components. Various secretome-based phenotypic screening methods facilitate the identification of targeted drugs and drug discovery. The library of secretome proteins has numerous advantages for target discovery, including the ability to directly identify active proteins by screening the library. It facilitates rapid comprehension of the disease pathway and subsequent formulation of hypotheses for drug discovery [78].

**Figure 4:**
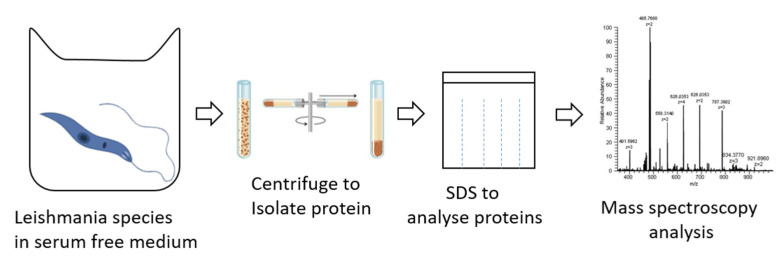
Secreted protein analysis (1) *Leishmania* spp. cultured in serum free medium; (2) Centrifuge collected aliquot; (3) Obtained sediment used for the quantification of secreted proteins; (4) Result analyzed by MS.

During the advancement of the *Leishmania* condition, the *Leishmania* parasite secretes a protein required for parasite survival and for disease pathogens to establish infection and modify the immune system inside the host [37]. Consequently, diverse mechanisms have been employed to identify secreted proteins under diverse conditions or stages of parasite development, as shown in Figure 4. Therefore, in this section, we attempt to review studies that provide pertinent information regarding the identification of secreted proteins in the various developmental stages of leishmaniasis.

Pissara and his co-workers examined seven *Leishmania* species in vitro without serum, and later they examined secreted proteins in seven *Leishmania* species during the promastigote stage using tandem mass spectrometry. As a result, it was discovered that the shared core secretome of all known *Leishmania* species resembles one-third of all secreted proteins. This indicated a preserved adaptation mechanism of the vertebrate host. Later, in bioinformatics analysis, it was found that most of the proteins were secreted through an unconventional mechanism. The high preservation of the protein and its recognized function in the *Leishmania* secretome data sets further validate the recommended use of promastigote-secreted proteins as a generator for antigen discovery and the production of vaccines and drug targets [79].

*Leishmania infantum*, which was grown in vitro in without serum RPMI-1640 medium, was examined, and secretions of *L. infantum* promastigote were obtained. Later, after lyophilization and deionization, 2D gel electrophoresis was performed to separate the proteins, followed by western blotting. This aided in the identification of nine proteins, the majority of which were involved in metabolic pathways, parasite survival and pathogenicity in *Leishmania* parasites: immune inhibitor A, phospholipase C, single peptide binds to chain and chitin binding protein. Selnomethionine crystal structure was observed in the secretion of *L. infantum* promastigotes. These proteins represent potential diagnostic and therapeutic targets for canine visceral leishmaniasis. *Leishmania* promastigotes’ secreted antigens are potential stimulants of the host immune system [80].

Silverman and his co-workers performed in vitro analysis to grow the *Leishmania donovani* in conditioned medium in order to analyze the stationary phase of the promastigote stage and isolate the secreted proteins through quantitative mass spectroscopy. As a result, 151 secreted proteins were identified by *L. donavani*. Later, bioinformatics techniques were applied that showed proteins were possibly transferred through the classic amino acid terminal secretion signal pathways. *Leishmania* conditioned medium had a predominance of known eukaryotic exosomal proteins, indicating the presence of a vesicle-based secretion system. Moreover, this result demonstrated that *L. donovani* protein secretion is a heterogeneous process that is unlikely to be regulated by a conventional amino-terminal signal. *L. donovani* may employ multiple non-classical secretion pathways, including the release of micro vesicles resembling exosomes [81].

**Table 1:** Summary of studies illustrated the type of protein, the drug against the target and the drug design method used in Leishmaniasis for distinct species.

Leishmania Species	Target	UniProt ID/PDB ID	Drug Design Approach	Designed Drug Molecule	References
L. *major*	Pteridine reductase 1(PTR1)	Q01782/2bfo	Molecular docking	Dihydropyrimidine	[82]
L. *infantum*	NADH dehydrogenase 2 (*Li*NDH2)	4g6g, 4g73	Homology modelling	6-methoxy-quinalidine	[83]
L. *donavani*	Pteridine reductase 1(PTR1) and dihydrofolate reductase-thymidylate synthase enzyme (DHFR-TS)		Molecular docking	Withaferin-A	[84]
L. *major*	Glycylpeptide n-tetradecanoyltransferase	4cgn	HTS	2-(4-fluorophenyl)-N-(3-piperidin-4-yl-1H-indol-5-yl)ethanamide (di diastereoisomer	[85]
*Leishmaniasis* spp.	Inositol phosphorylceramide synthase (IPCS)		Strcture based drug desiging	3-(1,3-Benzodioxol-5-yl)-6-{[(1E)-2	[86]
*Leishmaniasis* spp.	Trypanothione reductase	2JK6	Strcture based drug desiging	5-Nitrothiophene-2-carboxamides	
L. *donavani*	*Leishmania* Sperimidine synthase	3bwb	Molecular dynamic simulation	4-{[(2R)-2-[(2-oxo-4-phenyl-2 H-chromen-7-yl) oxy] propanamide] methyl}pyrdine-1-ium)., (1r, 4r)-4-{[4-(azaniumylmethyl)-1H-1, 2, 3-triazol-1-yl] methyl}-1-({ \2-oxo-1-azatricyclo [7.3.1.05.13] trideca-3,5,7,9 (13)-tetraen-4-yl} methyl) piperidin-1-ium).	[87]

## 6. Advanced Proteomics Approaches in Leishmaniasis Drug Discovery

Some of the advanced techniques such as LC-MS, MRM, SRM and SWATHE have also been explored in the discovery of drugs for leishmaniasis. In recent studies Kalpana Pai and his co-workers have used the SWATH-MS approach to analyze the antileishmanial effect of Commiphora wightii- Guggul and amphotericin B on a clinical isolate of Leishmania donovani. They showed that there is a considerable difference in the proteome profiles of drug sensitive and drug resistance groups, therefore highlighting the possibilities of the development of therapeutic antileishmanials [88]. Roseboom and his co-workers showed that HPLC-MS/MS could be successfully used for the quantification of the anti-leishmanial drug miltefosine in human skin tissue for its potential efficacy [89]. Nevertheless, the pharmacokinetic studies, which are key to the later development and validation of drugs, have also been conducted for leishmaniasis [90]. It is apparent that quantitative proteomics have also permeated into the pharmacokinetic studies and advanced the field of drug development for leishmaniasis. West and his co-workers successfully demonstrated that mass spectrometry-based proteomics could be used for identifying the protein target(s) of drug molecules with parallel thermodynamic measurements of protein-folding reactions in complex biological mixtures to detect protein–drug interactions. With this, they were able to identify yeast protein targets of the immunosuppressive drug cyclosporin A (CsA). Two of the ten protein targets identified in this proof of principle work were cyclophilin A and UDP-glucose-4-epimerase, both of which are known to interact with Cs, which could be an excellent model study for leishmaniasis drug discovery [91]. Veras and his co-workers have used unique approaches for anti-leishmaniasis drugs. They used the proteomic approach to search for new chemotherapeutic leishmanial targets and to modulate these targets to control *Leishmania* infection. In particular, the CBA mouse model of cutaneous leishmaniasis known for high susceptibility to infection in vivo was used and could successfully identify HSP90 and Hif-1a as potential targets [92].

## 7. Genomic and Transcriptomic Studies on Leishmaniasis Drug Discovery

Genomic and transcriptomic studies are used to understand the biology of *Leishmania* and provide information about the complex interaction occurring within the parasite–host–vector triangle [93]. The study conducted by Blackwell and his co-workers defines three omics studies on visceral leishmaniasis caused by *L. donavani*, including: (i) a genetic study in which various studies demonstrated that the most potent genetic factors for VL were the core of producing CD4+ T cell responses and that more comprehensive mapping and operational experiments provide high-resolution molecular insight into the interaction among parasite antigenic epitopes and HLA class II antigen-presenting cell molecules that influence immune responses toward disease-associated response; and (ii) a transcriptomic analysis revealing pathways important in Amphotericin B-treated vs. active VL cases, including evidence that anti-interleukin-10 triggers a storm of chemokines and cytokines in whole blood responses to soluble *Leishmania* antigens in productive cases. In general, our omics-based approaches validate that global analyses of genetic risk factors, host responses to infection, and the communication between host, parasite and the microbiome can identify the most significant factors that regulate the outcome of an infection [94]. The latest accomplishments of gene-expression and transcriptome analyses of *Leishmania spp*. allow us to compare the lifecycle stages of *Leishmania* to evaluate the different strains and species of *Leishmania* and their natural niche field, and also to compare their gene expression profiles between parasites and hosts [95]. Besides conventional approaches, emerging next generation genomics tools such as NGS, RNA-seq and Cos-Seq have recently been extensively used in leishmaniasis research. Dillon and his co-workers used RNA-seq to conduct high-resolution transcriptomic analysis to observe the gene expression and RNA processing that occurs as *L. major* transforms from the ineffective procyclic promastigote to an effective metacyclic promastigote, which shows the gene and process involved in the transition between stages as the parasite becomes capable of infecting mammalian host cells. It also provides evidence for hundreds of previously unknown gene functions. In addition, they discovered precise 5′ and 3′ UTR boundaries for most Leishmania genes, as well as extensive alternative trans-splicing and polyadenylation. This research builds on and improves existing expression datasets and provides a significantly more detailed view of the biology of *L. major*, which will inform the field and may provide a stronger foundation for drug discovery and vaccine development efforts [96]. Cos-Seq has recently been used for high-throughput identification of drug target and resistance mechanisms in the protozoan parasite *Leishmania*. It is functional cloning coupled to next-generation sequencing by using cosmid vector, and it is hence named Cos-seq. As an interesting outcome, the authors revealed more than sixty loci in the Leishmania genome that were enriched via drug selection with methotrexate and five major antileishmanials including antimony, miltefosine, paromomycin, amphotericin B and pentamidine, suggesting the prospects of this emerging technique and hastening the process of drug discovery and evaluation of drug-resistance [97]. The Mut-seq (mutation-sequencing) strategy is yet another prospective genomic technique for drug development. Bhattacharya and his co-workers have shown that conventional genomic screening does not reveal SNPs to be responsible for drug-resistance and associated principles; hence, Mut-seq could provide valuable information. They revealed associations between genes linked with lipid metabolism and resistance to MIL, and highlighted the role of a protein kinase in translation leading to resistance to PMM, showing the strengths of Mut-seq and its promise for future [98]. 

## 8. Conclusions and Future Perspectives

In this review, we attempted to assess how omics studies support drug discovery and their development via elucidating and applying host–pathogen interaction information with the data regarding proteins present at various stages of *Leishmania* infection. Here, we have categorized various sections of the review that discuss targeted protein studies in *Leishmania* parasites, protein identification during host-pathogen interactions at various stages of the *Leishmania* infection lifecycle, and protein identification during macrophage infection. These studies enrich our understanding of potential drug targets, the development of new drugs, and possible *Leishmania* treatments. Omics studies are also used in elucidating drug action, toxicity of drugs, drug efficacy and resistance under certain conditions. As proteins are the primary drug targets in disease states, more studies focused on proteomic analysis are needed. It is evident that proteomics techniques have largely improved in the past decade and the emergence of label-free and quantitative methods has become more precise and highly reproducible than conventional approaches, adding value to their potential applications in drug discovery. Nevertheless, promise alone is not sufficient to provide a global picture and, unable to define drug-resistance properties to a greater depth, genomics and transcriptomics shall augment the process of drug discovery in future. Reduction in cost, increase in coverage and the speed at which genomic and transcriptomic methods can be conducted today shall certainly lead to valuable inputs in leishmaniasis drug development in future. The use of advance proteomics approaches such as SWATHE, MRM, etc. and advanced genomics approaches such as cos-seq and mut-seq in leishmaniasis drug discovery have certainly pushed the frontiers forward. However, in view of the complexity of draggability and suitability of drugs for a disease, mere identification of drug targets is less useful than a much-extended use of omics approaches for further filtering of ligand–target pairs. Moreover, it is now believed that integrated omics approaches involving convergence of proteomics, genomics and transcriptomics hold promise for future drug development for leishmaniasis. 

## Data Availability

Not applicable.

## Supplementary Materials

The following supporting information can be downloaded at: https://www.mdpi.com/article/10.3390/pathogens12010039/s1, Table S1: Differentially regulated protein in L. inafntum amastigote and promastigote form; Table S2: Differentially regulated protein in L. mexicana amastigote and promastigote form; Table S3: Uniquely expressed proteins in L. infantum amastigote form; Table S4: Differentially expressed protein; Table S5: Up-regulated expressed proteins in L. major; Table S6: Up-regulated expressed proteins in L. tropica in; Table S7: Proteins differentially expressed in the amastigote form of L. infantum in KEGG pathway; Table S8: Differentially expressed proteins in the L. infantum promastigote form which were accessed from STRING database.
