# Supplementary material for: Omics Approaches in Drug Development against Leishmaniasis: Current Scenario and Future Prospects
Source: Pathogens. 2022 Dec 26;12(1):39. doi: 10.3390/pathogens12010039 (PMC9866966; doi:10.3390/pathogens12010039)
Supplement: Supplementary file 1 [file pathogens-12-00039-s001.zip › pathogens-2103220-supplementary.pdf]

Supplementary Table S1. Differentially regulate protein in *L. inafntum* amastigote and promastigote form in Lynn et al (2003).

**Accession number (DB database)**	**Proteins**
LinJ13.1520	Hypothetical protein, conserved
LinJ14.0700	Fatty acid elongase
LinJ27.1630	Hypothetical protein, conserved
LinJ24.1700	Succinate dehydrogenase flavoprotein
LinJ16.1510	Paraflagellar rod protein 2C
LinJ29.1890	Paraflagellar rod protein 1D
LinJ35.2020	Hypothetical protein, unknown function
LinJ11.0600	3-Methylcrotonoyl-CoA carboxylase beta subunit
LinJ35.5360	Polyadenylate-binding protein 1
LinJ28.0520	Hypothetical protein, conserved
LinJ08.1010	Stress-induced protein, sti1
LinJ12.0665	Surface antigen protein 2
LinJ30.0920	Surface protein amastin
LinJ09.0980	Calmodulin
LinJ24.0870	Triosephosphate isomerase
LinJ36.2330	Hypothetical protein, conserved
LinJ25.1670	Hypothetical protein, conserved
LinJ10.0490	GP63-1, leishmanolysin
LinJ17.1390	Translation initiation factor
LinJ17.0010	Hypothetical protein, conserved
LinJ31.3270	Hypothetical protein, conserved
LinJ31.2650	Ubiquinol-cytochrome-c reductase
LinJ18.1500	H1A-1P-type H+-ATPase
LinJ16.1390	Cytochrome c
LinJ20.1350	SMP-1, small myristoylated protein-1, putative
LinJ35.2270	KMPII-2, kinetoplastid membrane
Amastigote	
LinJ07.0060	Alpha-adaptin-like protein
LinJ13.0810	Hypothetical protein, conserved
LinJ35.1490	Arginase
LinJ30.1370	Hypothetical protein, unknown function
LinJ11.1170	Hypothetical protein, conserved
LinJ28.2430	Glycosomal membrane protein
LinJ17.0110	Elongation factor 1-alpha
LinJ29.0860	Cysteine peptidase C (CPC)
LinJ31.1240	Vacuolar-type proton translocating pyrophosphatase 1
LinJ16.0960	Sucrose-phosphate synthase-like protein
LinJ29.2310	GTP-binding protein
LinJ21.1170	Histone H2A
LinJ30.3000	Glyceraldehyde 3-phosphate dehydrogenase, glycosomal
LinJ21.0310	Hexokinase

LinJ35.1220	Hypothetical protein, conserved
LinJ16.0560	Orotidine-5-phosphate decarboxylase
LinJ29.2620	ATP-dependent phosphofructokinase
LinJ35.0680	Hypothetical protein, conserved
LinJ15.1120	TRYPtryparedoxin peroxidase
LinJ27.1900	FtsJ cell division protein
LinJ07.1020	Splicing factor ptsr1-like protein
LinJ25.1790	Pyruvate dehydrogenase E1 beta subunit
LinJ33.0300	Hypothetical protein, conserved

Supplementary Table S2. Differentially regulate protein in *L. mexicana* amastigote and promastigote form in Lynn et al (2003).

**Accession number (DB database)**	**Proteins**
LinJ33.1030	Hypothetical protein, conserved
LinJ09.0950	Polyubiquitin
LinJ16.0750	Hypothetical protein, unknown
	function
LinJ21.0310	Hexokinase, putative
LinJ32.0410	ATP-dependent RNA helicase, putative
LinJ11.1000	Pyruvate phosphate dikinase, putative
LinJ35.2270	KMPII-2kinetoplastid membrane
	protein-11

Supplementary Table S3. Uniquely expressed proteins in *L. infantum* amastigote form in McNicoll et al. 2006.

Proteins
Hypothetical protein, unknown function
Hypothetical protein, conserved
ATP-dependent DEAD/H RNA helicase,
Hypothetical protein, unknown function
Hypothetical protein, conserved
Seryl-tRNA synthetase, putative
Carboxypeptidase, putative
Pyrroline-5-carboxylate reductase
Myo-inositol-1-phosphate synthase
Thiol specific antioxidant
Heat-shock protein, putative
Peptidylprolyl isomerase-like protein
La RNA binding protein, putative
Hypothetical protein, conserved
Acetyl-CoA synthetase, putative
Hypothetical protein, unknown function
Protein disulfide isomerase, putative
6-Phosphogluconolactonase
Heat-shock protein hsp70, putative
Aspartyl aminopeptidase, putative
Hypothetical protein, unknown function
Heat-shock 70-related protein 1, t.
TPR domain protein, conserved
Cytoskeleton-associated protein CAP5.5
Prostaglandin f2-alpha synthase
Calreticulin, putative
Hypothetical protein, conserved
Chaperonin containing t-complex protein,
Hypothetical protein, conserved
Proteasome regulatory non-ATPase sub., putative
3-Hydroxyisobutyryl-coenzyme a hydrolase-like
Heat-shock protein 83-1
Mitogen activated protein kinase, putative
Peptidase M20/M25/M40, putative
Glucose-6-phosphate dehydrogenase
d-Isomer specific 2-hydroxyacid dehydrogenase
Hypothetical protein, unknown function
Hypothetical protein, unknown function
Hypothetical protein, conserved
Proteasome activator protein pa26, putative
Fructose-1,6-bisphosphate aldolase

Transitional endoplasmic reticulum
ATPase, putative
Chaperonin Hsp60, Mitochondrial precursor

Supplementary Table S4. Differentially expressed protein of Leifso et al. (2007) study.

Accession number (DB database)	Protein name
LinJ21.0350	Hypothetical protein, conserved
LinJ36.4560	Chaperonin HSP60, mitochondrial
LinJ21.0140	Precursor hexokinase, putative
LinJ02.0380	Hypothetical protein, conserved
LinJ32.3680	Ribosomal protein l3, putative
LinJ27.1480	Hypothetical protein, unknown function
LinJ10.1330	Histone h3
LinJ13.0090	Carboxypeptidase, putative
LinJ27.0150	Methylmalonyl-coenzyme a mutase, putative
LinJ04.0970	60S ribosomal protein L10, putative
LinJ30.4300	60S ribosomal protein L9, putative
LinJ36.1640	40s ribosomal protein s10, putative
LinJ19.1440	Peptidylprolyl isomerase-like protein
LinJ32.0770	ATP-dependent RNA helicase, putative
LinJ31.2550	Prostaglandin f2-alpha synthase
LinJ25.1040	Hypothetical protein, conserved
LinJ35.3330	Cystathione gamma lyase, putative
LinJ36.2470	Elongation factor 2
LinJ06.0010	Histone h4
LinJ21.1910	Histone H4
LinJ17.0500	Hypothetical protein, conserved
LinJ18.0220	RNA-binding protein, putative
LinJ22.1590	Hypothetical protein, unknown function
LinJ30.0670	Kinesin, putative
LinJ24.1500	Hypothetical protein, conserved
LinJ07.1050	RNA binding protein-like protein
LinJ30.2810	Heat shock 70-related protein 1, mitochondrial precursor, putative
LinJ36.1010	Dihydrolipoamide acetyltransferase precursor, putative
LinJ35.0510	40S ribosomal protein S3a, putative
LinJ33.1290	Hypothetical protein
LinJ28.1430	Haloacid dehalogenase-like hydrolase, putative

LinJ26.1920	Mitotubule-associated protein Gb4, putative
LinJ21.0390	DNAJ protein, putative
LinJ06.0370	Glutamine synthetase, putative
LinJ26.1930	Hypothetical protein, conserved
LinJ33.2870	Hypothetical protein, unknown function
Li nJ19.0040	Histone H2B
LinJ19.0040	Histone H2B
LinJ36.4490	Phosphomannomutase, putative
LinJ28.2790	Hypothetical protein, conserved
LinJ28.0550	Ribosomal protein s26, putative
LinJ30.0330	3-Oxoacid CoA transferase 1
LinJ30.0630	Hypothetical protein, conserved
LinJ36.2700	Hypothetical protein, conserved
LinJ07.1300	Hypothetical protein, conserved
LinJ08.1170	Beta tubulin
LinJ25.0730	Hypothetical protein, conserved
LinJ32.2360	ATP-dependent zinc metallopeptidase, putative
LinJ25.0780	Protein phosphatase, putative
LinJ32.3870	Dihydrolipoamide dehydrogenase, putative
LinJ13.0810	Hypothetical protein, conserved
LinJ14.0980	Immunodominant antigen, putative
LinJ25.1120	Hypothetical protein, conserved
LinJ23.0400	NADP-dependent alcohol dehydrogenase, putative
LinJ27.1680	Hypothetical protein, conserved
LinJ18.0740	Elongation factor TU, putative
LinJ05.0380	Microtubule-associated protein, putative
LinJ14.1250	Enolase
LinJ14.1250	Enolase

Supplementary Table S5. Up regulated expressed proteins in *L. major* in Ashrafmansouri et al. (2020).

Protein ID (STRING database)	Protein name
O62591	Probable eukaryotic initiation factor 4A
Q4QEB3	GMP reductase
Q4QG98	60S ribosomal protein L18
E9ABZ4	Putative carboxylase
E9ACG7	Putative delta-1-pyrroline-5-carboxylate dehydrogenase
E9AD07	Putative methylmalonyl-coenzyme a mutase
E9ADF9	Putative glycosomal phosphoenolpyruvate carboxykinase
E9ADX3	Tryparedoxin
E9AE57	Putative fumarate hydratase
E9AEU1	Putative NADH-dependent fumarate reductase
E9AFK3	Putative 60S ribosomal protein L23
E9AFP0	Polyadenylate-binding protein
Q07DU5	Peroxidoxin 2
Q4QF5	Dihydrolipoamide acetyltransferase component of pyruvate dehydrogenase complex
Q4Q11R4	Putative universal minicircle sequence binding protein
Q4Q259	Elongation factor 2
Q4Q2H7	Putative vacuolar ATP synthase catalytic subunit-A
Q4Q745	Putative heat shock 70-related protein 1, mitochondrial
Q4Q838	Putative glycosomal membrane protein
Q4Q9H4	Putative 60S ribosomal protein L7
Q4Q9M4	Succinate-CoA ligase (ADP-forming) subunit alpha, mitochondrial
Q4Q9R2	Polyprenolreductase
Q4Q9Y0	Putative cytochrome c oxidase VII
Q4QAP8	Triosephosphateisomerase
Q4QEM2	Paraflagellar rod protein 2C
Q4QEX4	Putative 60S ribosomal protein L21
Q4QF42	Putative nucleolar RNA binding protein
Q4QFG2	Putative 60S ribosomal protein L13a
Q4QFP8	Putative small myristoylated protein-3
Q4QG31	40S ribosomal protein S4
Q4QGN9	Glucose-6-phosphate isomerase
Q4QI59	Uncharacterized protein
Q4QJ88	V-type proton ATPase subunit
Q9XZX2	Putative ADP-ribosylation factor

Supplementary Table S6. Up regulate expressed proteins in *L. tropica* in Ashrafmansouri et al. (2020) study data access through STRING database

Protein ID	Proteins
E9AEB3	ATP-dependent 6-phosphofructokinase
E9AEX6	Putative reiske iron–sulfur protein
E9AF23	40S ribosomal protein S6
Q4Q5N9	Putative prostaglandin f synthase
Q4Q5P5	Putative ATP-dependent RNA helicase
Q4Q8V7	Putative 60S ribosomal protein L35
Q4Q9Y1	Aldehyde dehydrogenase, mitochondrial
Q4QBD7	Putative cytochrome c oxidase subunit 10
Q4QBH2	Peroxidoxin
Q4QCE1	Putative 60S ribosomal protein L36
Q4QDK0	ATP/ADP translocase
Q4QDX9	Putative 60S ribosomal protein L10a
Q4QEX6	Putative fucose kinase
Q4QIP1	Putative 60S ribosomal protein L7a
Q4QJF1	ATPase alpha subunit
Q4VT70	Phosphotransferase
Q9U0V9	Possible 3-ketoacyl-CoA thiolase

Supplementary Table S7. Fialho junior et al. (2021) describe a protein that is differentially expressed in the amastigote form of *L. infantum* in KEGG pathway

**Proteins**
ATPase beta subunit
Dihydrolipoamide dehydrogenase
Enolase
Fructose-1,6-bisphosphate aldolase
Glutamate dehydrogenase
Glyceraldehyde 3-phosphate dehydrogenase, glycosomal
Pyruvate dehydrogenase E1 component alpha subunit
Transaldolase
Triosephosphate isomerase
Tryparedoxin peroxidase
**Ribosome**
40S ribosomal protein S11
40S ribosomal protein S13
40S ribosomal protein S3
60S ribosomal protein L18a
60S ribosomal protein L6
60S ribosomal protein L9

Supplementary Table S8. Filaho junior et al. (2021) used the STRING database to access a differentially expressed protein in the *L. infantum* promastigote form.

**Proteins**
Molecular function (GO)
Nucleic acid binding
40S ribosomal protein S2
40S ribosomal protein S3, putative
60S ribosomal protein L5, putative
ATP-dependent RNA helicase
Conserved hypothetical protein LinJ.13.0270
Conserved hypothetical protein LinJ.27.1220
Eukaryotic initiation factor 4a, putative
Histone H2A
Histone H3
Histone H3 variant, putative
RNA-binding protein, putative
RNA helicase
RNA-binding protein, putative, UPB1
RNA-binding protein, putative, UPB2
Cellular component (GO)
Nucleo
Histone H2A
Histone H3
Histone H3 variant, putative
U3 small nucleolar ribonucleoprotein protein MPP10
KEGG pathways
Metabolic pathways
2,3-Bisphosphoglycerate-independent phosphoglycerate mutase
3-Ketoacyl-CoAthiolase
ATPase beta subunit
Dihydrolipoamide dehydrogenase

Enolase
Fructose-1,6-bisphosphate aldolase
Hexokinase
Isocitrate dehydrogenase [NADP], mitochondrial precursor
Orotidine-5-phosphate decarboxylase/orotate phosphoribosyltransferase
Peroxidoxin
Phosphomannomutase
Pyruvate dehydrogenase E1 beta subunit
Reiske iron-sulfur protein precursor
S-adenosylhomocysteine hydrolase
Spermidine synthase
Succinyl-CoA ligase [GDP-forming] beta-chain
Trypanothione reductase
Tryparedoxin peroxidase
UDP-glucose pyrophosphorylase
Vacuolar ATP synthase subunit b
Ribosome
40S ribosomal protein S12
40S ribosomal protein S2
40S ribosomal protein S27-1
40S ribosomal protein S3
40S ribosomal protein S6
60S ribosomal protein L19
60S ribosomal protein L19
60S ribosomal protein L23
60S ribosomal protein L27A/L29
60S ribosomal protein L36
60S ribosomal protein L6
Ribosomal protein L3
Oxidative phosphorylation

ATPase beta subunit
Reiske iron-sulfur protein precursor
Vacuolar ATP synthase subunit b
Vacuolar-type proton translocating pyrophosphatase 1
